# Genetic evolution of hemagglutinin and neuraminidase genes of H5N1 highly pathogenic avian influenza viruses in Thailand

**DOI:** 10.7717/peerj.14419

**Published:** 2022-11-30

**Authors:** Pirom Noisumdaeng, Juthamas Phadungsombat, Sasrinakarn Weerated, Witthawat Wiriyarat, Pilaipan Puthavathana

**Affiliations:** 1 Faculty of Public Health, Thammasat University, Khlong Luang, Pathum Thani, Thailand; 2 Thammasat University Research Unit in Modern Microbiology and Public Health Genomics, Thammasat University, Khlong Luang, Pathum Thani, Thailand; 3 Mahidol-Osaka Center for Infectious Diseases (MOCID), Faculty of Tropical Medicine, Mahidol University, Bangkok, Thailand; 4 Department of Viral Infections, Research Institute for Microbial Diseases, Osaka University, Osaka, Japan; 5 Faculty of Veterinary Science, Mahidol University, Nakhon Pathom, Thailand; 6 Center for Research and Innovation, Faculty of Medical Technology, Mahidol University, Nakhon Pathom, Thailand

**Keywords:** H5N1 highly pathogenic avian influenza (HPAI) virus, Hemagglutinin, Neuraminidase, Genetic evolution, Reassortant H5Nx viruses

## Abstract

**Background:**

Ongoing outbreaks of H5N1 highly pathogenic avian influenza (HPAI) viruses and the emergence of the genetic-related hemagglutinin (*HA*) gene of reassortant H5Nx viruses currently circulating in wild birds and poultries pose a great global public health concern. In this study, we comprehensively analyzed the genetic evolution of Thai H5N1 *HA* and neuraminidase (*NA*) genes between 2003 and 2010. The H5N1 Thailand virus clade 2.3.4 was also genetically compared to the currently circulating clade 2.3.4.4 of H5Nx viruses.

**Methods:**

Full-length nucleotide sequences of 178 *HA* and 143 *NA* genes of H5N1 viruses circulating between 2003 and 2010 were phylogenetically analyzed using maximum likelihood (ML) phylogenetic construction. Bayesian phylogenetic trees were reconstructed using BEAST analysis with a Bayesian Markov chain Monte Carlo (MCMC) approach. The maximum clade credibility (MCC) tree was determined, and the time of the most recent common ancestor (tMRCA) was estimated. The H5N1 HA nucleotide sequences of clade 2.3.4 Thailand viruses were phylogenetically analyzed using ML phylogenetic tree construction and analyzed for nucleotide similarities with various subtypes of reassortant H5Nx HA clade 2.3.4.4.

**Results:**

ML phylogenetic analysis revealed two distinct HA clades, clade 1 and clade 2.3.4, and two distinct NA groups within the corresponding H5 clade 1 viruses. Bayesian phylogenetic reconstruction for molecular clock suggested that the Thai H5N1 HA and NA emerged in 2001.87 (95% HPD: 2001.34-2002.49) and 2002.38 (95% HPD: 2001.99-2002.82), respectively, suggesting that the virus existed before it was first reported in 2004. The Thai H5N1 HA clade 2.3.4 was grouped into corresponding clades 2.3.4, 2.3.4.1, 2.3.4.2, and 2.3.4.3, and shared nucleotide similarities to reassortant H5Nx clade 2.3.4.4 ranged from 92.4-96.8%. Phylogenetic analysis revealed monophyletic H5Nx clade 2.3.4.4 evolved from H5N1 clade 2.3.4.

**Conclusion:**

H5N1 viruses existed, and were presumably introduced and circulated in avian species in Thailand, before they were officially reported in 2004. *HA* and *NA* genes continuously evolved during circulation between 2004 and 2010. This study provides a better understanding of genetic evolution with respect to molecular epidemiology. Monitoring and surveillance of emerging variants/reassortants should be continued.

## Introduction

Influenza A viruses are classified into 18 hemagglutinin (HA) (H1-H18) and 11 neuraminidase (NA) (N1-N11) subtypes. Subtypes H1-H16 and N1-N9 can be isolated from aquatic birds, while H17N10 and H18N11 were discovered in bats using nucleotide sequence analysis (Webster et al., 1992; Dugan et al., 2008; Forrest & Webster, 2010; Tong et al., 2012; Tong et al., 2013; Long et al., 2019). Several subtypes of avian influenza A viruses, *i.e.*, H5N1, H5N6, H5N8, H7N7, H7N9, and H9N2, have been reported to cross the species barrier and infect humans (Wong & Yuen, 2006; Peiris, de Jong & Guan, 2007; Forrest & Webster, 2010; Gao et al., 2013; Mok et al., 2015). Among these avian influenza subtypes, H5N1 highly pathogenic avian influenza (HPAI) virus is the most virulent, causing the highest percent case fatality (53%) in humans (World Health Organization , 2022). The recent emergence of evolutionary-related H5 HA of reassortant H5Nx subtypes in combination with different NA subtypes has been globally detected in wild birds and poultries (Li et al., 2017; Antigua et al., 2019; Nuñez & Ross, 2019; Liang et al., 2021; Li, Su & Smith, 2021; Gu et al., 2022).

The first emergence of H5N1 viruses causing human infections occurred in Hong Kong in 1997 and resulted in 18 cases and six deaths (33% case fatality) (Gutiérrez et al., 2009). The virus re-emerged in 2003, and it has been uncontrollable until the present. Between January 2003 and June 2022, there have been 864 human cases including 456 fatalities globally (53% case fatality) (World Health Organization , 2022). The latest case of H5N1 infection was reported in the United States in April 2022 (Centers for Disease Control and Prevention , 2022; World Health Organization , 2022). Moreover, H5Nx reassortants with different NA subtypes, particularly H5N6 and H5N8, emerged in China during the successful control of H5N1 virus in poultry (Li, Su & Smith, 2021), and subsequently caused global outbreaks, mainly in Asia, Europe, and North America (Li et al., 2017; Antigua et al., 2019; Nuñez & Ross, 2019; Liang et al., 2021; Li, Su & Smith, 2021; Gu et al., 2022). A total of 78 laboratory-confirmed H5N6 human cases, 32 of which with fatal outcomes (41% case fatality), were reported in China from 2015–2022 and Laos in 2021 (World Health Organization , 2022), while seven positive cases of H5N8 virus infection were reported in Russia in 2021 (World Health Organization , 2021a). Thailand first reported an H5N1 avian influenza outbreak in poultry and humans in January 2004 (Puthavathana et al., 2005). The last human case was reported in 2006, while the last poultry outbreak was reported in 2008 (Uchida et al., 2008; Chaichoune et al., 2009). Nevertheless, three new genomic sequences of H5N1 isolates were deposited in the GenBank database in 2010. There has been a total of 25 human cases with 17 deaths (68% case fatality). To date, no H5Nx human infections have been detected in Thailand, although one isolate of the H5N8 virus was found in chicken in 2008 (World Health Organization, 2014).

A putative ancestor of H5N1 viruses re-emerged in 2003 is A/goose/Guandong/1/96 (H5N1) (Gs/GD/1/96) (WHO/OIE/FAO H5N1 Evolution Working Group , 2008; Harfoot & Webby, 2017). All the viral genomic segments are of avian origin (World Health Organization Global Influenza Program Surveillance Network, 2005) and naturally acquired through genetic reassortment. The *HA* and *NA* genes were derived from the Gs/GD/1/96-like lineage; while six internal genes originated from various avian influenza virus subtypes, serving as the basis for the assignment to different genotypes based on the gene-constellation analysis of each genomic segment using phylogenetic analysis (neighbor-joining bootstrap support >70% or Bayesian posterior probability >95%) (Guan et al., 2002; Gutiérrez et al., 2009; Li et al., 2004). By 2001, six genotypes (A, B, C, D, E and X_0_) had been identified, and an additional nine new genotypes (G, V, W, X1, X2, X3, Y, Z and Z ^+^) were detected between 2002 and 2004. Genotype Z has become the dominant H5N1 virus in southern China and has been responsible for subsequent outbreaks in Asia (Gutiérrez et al., 2009). Continuous H5N1 virus outbreaks and the emergence of reassortant H5Nx viruses over the past decade have resulted in the evolution and genetic diversity of H5 HA. The WHO/OIE/FAO H5N1 Evolution Working Group identified and updated the nomenclature for genetic clades (clades 0-9) based on HA nucleotide sequences (WHO/OIE/FAO H5N1 Evolution Working Group , 2008; World Health Organization/World Organisation for Animal Health/Food and Agriculture Organization, WHO/OIE/FAO)H5N1 Evolution Working Group(2014 Smith et al., 2015). With the rapid evolution of H5 HA, new clades have emerged in several countries, *e.g.*, clades 2.1.3.2a-c in Indonesia, Vietnam, and Cambodia (Le & Nguyen, 2014; Lee et al., 2015; Smith et al., 2015; Suttie et al., 2019). Furthermore, the widespread emergence of H5 HA clade 2.3.4.4 of Nx reassortants (including H5N1, H5N2, H5N3, H5N5, H5N6, and H5N8) has been documented in Asia, Europe, and North America (Li et al., 2017; Antigua et al., 2019; Liang et al., 2021; Li, Su & Smith, 2021; Gu et al., 2022). The currently circulating H5 clade 2.3.4.4 was further classified into clades 2.3.4.4a-h after a proposed update to the unified nomenclature for HPAI H5 viruses by WHO (World Health Organization , 2021b). H5N1 genotypes Z and V, and HA genetic clades 1 and 2.3.4 were identified in Thailand between 2003 and 2010 (Chutinimitkul et al., 2007; Chaichoune et al., 2009; Amonsin et al., 2010), and since then they have not been detected till present.

In this study, we comprehensively analyzed the genetic evolution of Thai H5N1 HA and NA between 2003 and 2010. The H5N1 Thailand virus clade 2.3.4 was also genetically compared to the currently circulating clade 2.3.4.4 of H5Nx viruses.

## Material and Methods

### H5N1 HA and NA genomic sequences and data sets

HA and NA nucleotide sequences from 333 H5N1 viruses reported in Thailand between 2003 and 2010 were retrieved from the Influenza Virus Resource (National Center for Biotechnology Information, U.S. National Library of Medicine) (https://www.ncbi.nlm.nih.gov/genomes/FLU/Database/nph-select.cgi?go=database). Among them, full-length nucleotide sequences of 178 HA and 143 NA were retrieved and phylogenetically analyzed (Tables S1 and S2).

### Phylogenetic analysis of HA and NA

Full-length of 178 HA and 143 NA nucleotide sequences were aligned using Muscle in AliView v1.26 (https://ormbunkar.se/aliview/) (Larsson, 2014) with the reference sequences corresponding to clades 0, 1, 2.1.1, 2.1.2, 2.1.3, 2.2, 2.2.1, 2.2.2, 2.3.1, 2.3.2, 2.3.3, 2.3.4, 3, 4, 5, 6, 7, 8, and 9 reported from World Health Organization/World Organisation for Animal Health/Food and Agriculture Organization (WHO/OIE/FAO) H5N1 Evolution Working Group (WHO/OIE/FAO H5N1 Evolution Working Group , 2008; World Health Organization , 2014; Smith et al., 2015). For genetic clade classification, a maximum likelihood (ML) phylogenetic tree was constructed using IQ-TREE with 1,000 ultrafast bootstrap replicates with substitution model of TIM+F+G4 and GTR+F+G4 which were the best-fit models for HA and NA alignment, respectively (Trifinopoulos et al., 2016). The percentages of bootstrapping with ≥80 in which the associated taxa clustered together were shown on the nodes. The datasets of viruses analyzed in the study are shown in Tables S1 and S2.

To determine divergence time and ancestral origin, datasets including ancestor strain Gs/GD/1/96 (GenBank accession number: AF148678) and H5N1 viruses reported in Thailand and neighboring countries (Vietnam, Cambodia, China, Hong Kong, Laos, and Malaysia) during 2002 and 2014 were prepared (Data S1). The time-scaled tree was reconstructed using BEAST package v1.10.4 with a Bayesian Markov chain Monte Carlo (MCMC) approach under GTR substitution model, strict clock, and exponential growth tree prior (Suchard et al., 2018). The triplicate runs of MCMC lengths of 30,000,000 generations with sampling every 3,000 generations were performed and the individually obtained effective sample sizes over 200 traced in Tracer v1.7.1 were combined in LogCombiner v1.10.4 provided in the BEAST package. The maximum clade credibility (MCC) tree was determined using TreeAnnotator v1.10.4 and visualized in Figtree v1.4.4 (http://tree.bio.ed.ac.uk/software/figtree). The time of the most recent common ancestor (tMRCA) and its 95% highest probability density (95% HPD) were expressed as a year.

### Genetic comparison between H5N1 clade 2.3.4 and H5Nx clade 2.3.4.4

H5N1 HA nucleotide sequences of identified clade 2.3.4 Thailand viruses were phylogenetically analyzed using ML phylogenetic tree construction with the reference strains including H5N1 HA clades 2.3.4, 2.3.4.1, 2.3.4.2, 2.3.4.3 (covering 2005 to 2013) and various subtypes of reassortant H5Nx HA clades 2.3.4.4 and 2.3.4.4a-h covering 2013 to 2021 (World Health Organization , 2021b), retrieved from GenBank and the EpiFlu™ database of the Global Initiative on Sharing All Influenza Data (GSAID) database (https://www.gisaid.org/) (Shu & McCauley, 2017) (Data S1). The nucleotide similarity was analyzed using the Sequence Identity Matrix application on BioEdit program v7.0.5.2 (Hall, 1999).

## Results

### Genetic characteristics of Thai H5N1 HA and NA

As of 30 September 2021, Thailand had submitted a total of 333 H5N1 isolates to the Influenza Virus Resource (NCBI). There was one isolate collected in 2003, 198 isolates in 2004, 81 isolates in 2005, 22 isolates in 2006, 11 isolates in 2007, 17 isolates in 2008, and three isolates in 2010. The viruses were discovered from several host species *i.e.*, chickens (*n* = 162), ducks (*n* = 37), birds (*n* = 93), humans (*n* = 20), tigers (*n* = 11), leopards (*n* = 6), cats and canines (*n* = 2), the environment, and unknown sources (*n* = 2). Among the 333 H5N1 viruses, there were 347 HA nucleotide sequences of which 178 full-length HA sequences were available. While there were 318 NA nucleotide sequences, only 143 full-length NA sequences were reported in the database. The genetic determinants on HA and NA were analyzed. All viruses contained multiple basic amino acids (mostly RE RRRKKR↓GLF [81%]) at the HA cleavage site, and showed *α*2,3-galactose linked-sialic acid avian type receptor preference (residues 190E [100%], 225G [100%], 226Q [100%], and 228G [100%]) (H3 numbering) on HA molecules. In addition, the 20-amino acid deletion (100%), and oseltamivir susceptible marker (274H, 100%) (N2 numbering) were present in NA of all viruses (Table 1).

**Table 1:** Analysis for genetic determinants of HPAI H5N1 HA and NA compared to the H5N1 genetic change inventory (Centers for Disease Control and Prevention, 2012).

**Protein**	**Amino acid observed in Thai HPAI H5N1 isolates**	**Amino acid mutation previously reported**	**Association and function**
HA^a^	190E (100%) and 225G (100%)	D190E and D225G	190D and 225D - human receptor preference 190E and 225G - avian receptor preference
	226Q (100%) and 228G (100%)	Q226L and G228S	226Q and 228G - receptor binding site for avian receptors 226L and 228S - receptor binding site for human receptors
	RE RRRKKR↓GLF (81%) RE KRRKKR↓GLF (10%) IE RRRKKR↓GLF (4%) RE RKRKKR↓GLF (3%) RE RRRKR↓GLF (1%) RE RRRKR↓GLF (1%)	RRRKK (329–333)	Polybasic amino acid insertion at HA cleavage site: RRRKK - indicator for HPAI and systemic infection
NA^b^	20-amino acid deletion at stalk region (100%)	20-amino acid deletion at stalk region^c^	Contributes to the high pathogenicity of H5N1 viruses
	274H (100%)	274H 274Y	Oseltamivir sensitive Oseltamivir resistance

**Notes.**

a Amino acid position on HA based on H3 numbering.

b Amino acid position on NA based on N2 numbering.

c NA of A/goose/Guangdong/1/96 (H5N1) contained CNQSIITYENNTWVNQTYVN at stalk region, but it was not present in NA of HPAI H5N1 Thailand isolates.

### ML phylogenetic analysis of Thai H5N1 HA and NA

**Figure 1:**
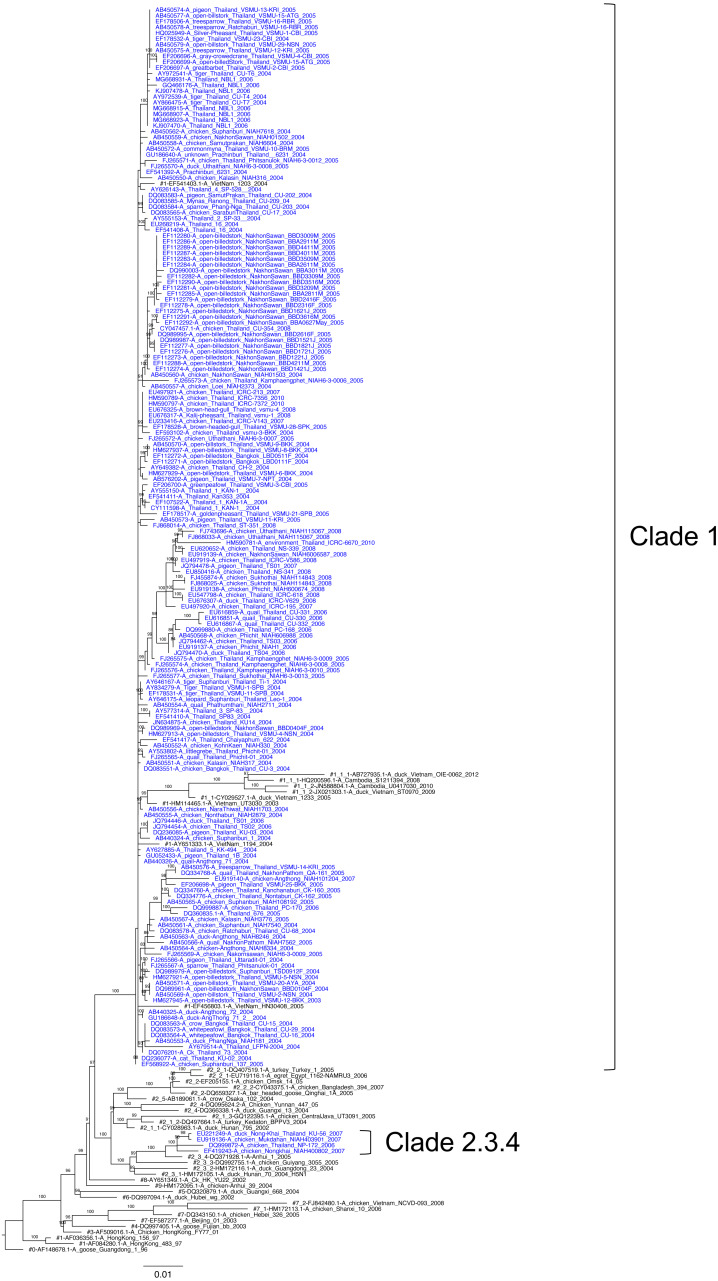
Maximum likelihood phylogenetic analysis for H5 HA clade identification among H5N1 isolates reported in Thailand between 2003 and 2010. The percentage of bootstrapping ( >80) in which the associated taxa clustered together is shown on the nodes. Thai H5N1 and reference viruses are shown in blue and black, respectively. The reference clades are shown with the sharp symbol (#) in the front of each tip. The ML tree was rooted to A/goose/Guangdong/1/96 (H5N1).

We phylogenetically analyzed the full-length 178 HA and 143 NA nucleotide sequences using ML phylogenetic construction together with the H5 clade reference sequences. The phylogenetic tree topology of H5 HA revealed two distinct clades (clade 1 and clade 2.3.4). A nucleotide similarity of 93.0–96.2% was revealed between two genetic clades, while a nucleotide similarity of 98.3–99.1% was revealed within intra-clade 1 viruses. Clades 1 and 2.3.4 viruses were clearly separated by 100% bootstrapping support (Fig. 1). In addition, clade 1 was the major clade containing viruses collected from 2003–2010, mostly during the early introduction time period 2003–2005, whereas clade 2.3.4 was the minor clade with only four viruses collected in 2006–2007 from the northeastern part of Thailand.

**Figure 2:**
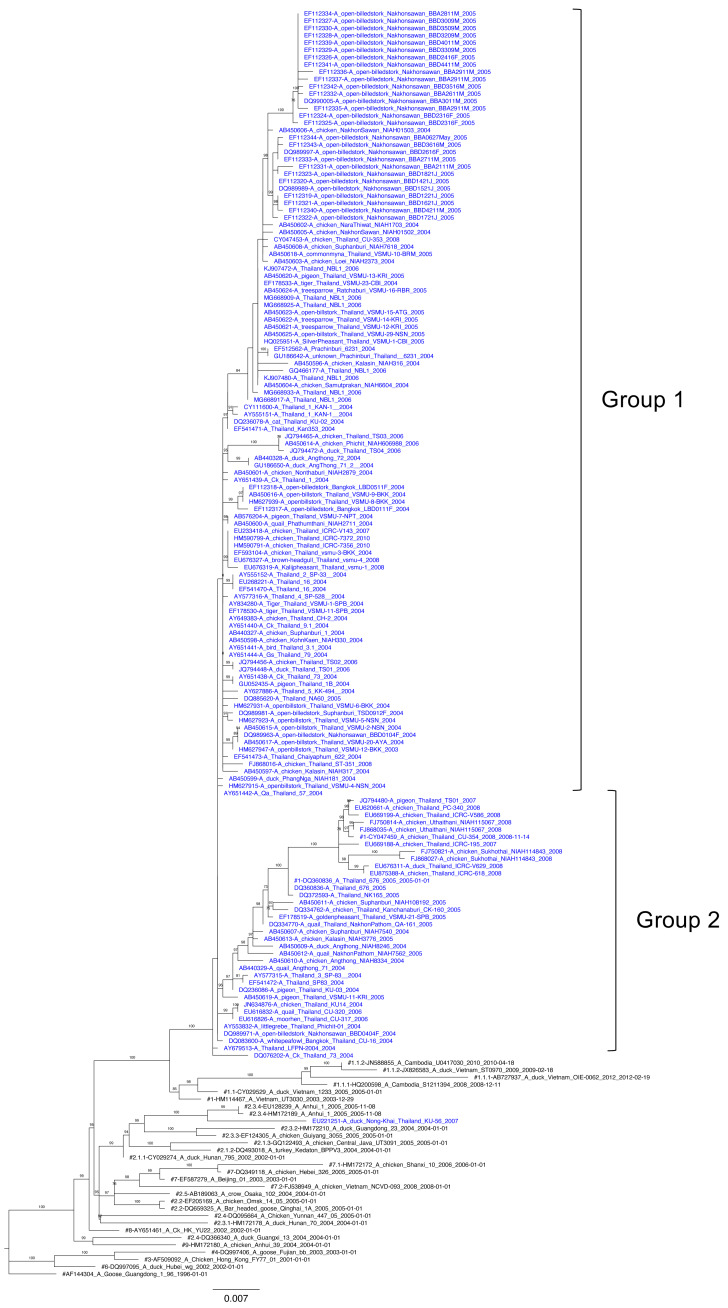
Maximum likelihood phylogenetic analysis of NA among H5N1 isolates reported in Thailand between 2003 and 2010. The percentage of bootstrapping ( >80) in which the associated taxa clustered together is shown on the nodes. Thai H5N1 and reference viruses are shown in blue and black, respectively. The reference clades are shown with the sharp symbol (#) in the front of each tip. The ML tree was rooted to A/goose/Guangdong/1/96 (H5N1).

For NA-based ML phylogenetic tree topology, H5N1 NA corresponding to H5 clade 1 virus was classified into two distinct major groups (groups 1 and 2). Most H5N1 viruses contained NA genes genetically related to viruses from Vietnam and Cambodia, except one isolate of clade 2.3.4 (A/duck/NongKhai/KU-50/2007, EU221251) which was closely related to viruses corresponding to clade 2.3.4 identified in China (Fig. 2). The nucleotide sequence similarity among NA ranged from 96.9–100%.

### Divergence time estimation by Bayesian phylogenetic analysis

**Figure 3:**
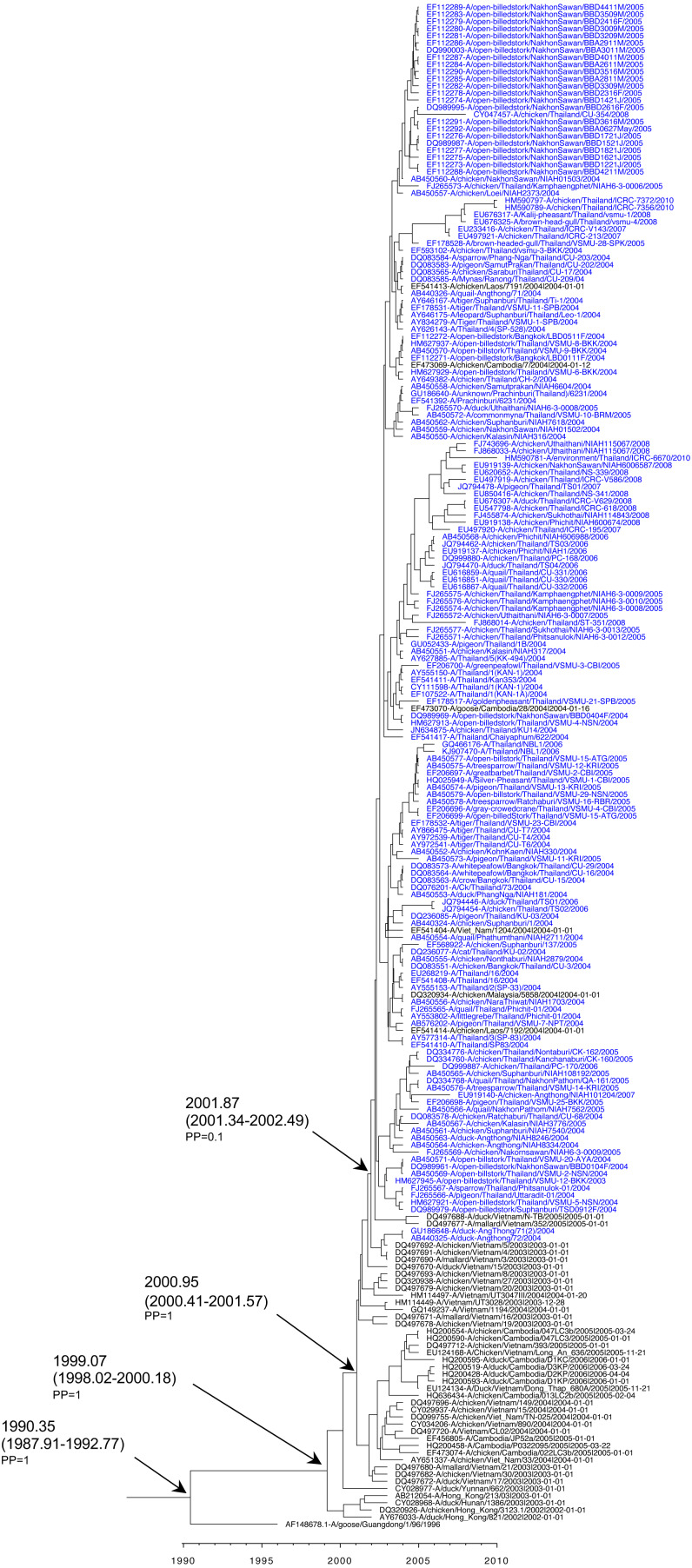
Maximum clade credibility tree based on HA of H5N1 clade 1 viruses. Time-scale tree was estimated in BEAST package v1.10.4 with GTR model, strict clock, and exponential growth tree prior. The tMRCA and 95% HPD are indicated with a black arrow, and posterior probability (PP) value is indicated. The name of each taxon is presented in order of GenBank accession number, virus name, and year of collection.

**Figure 4:**
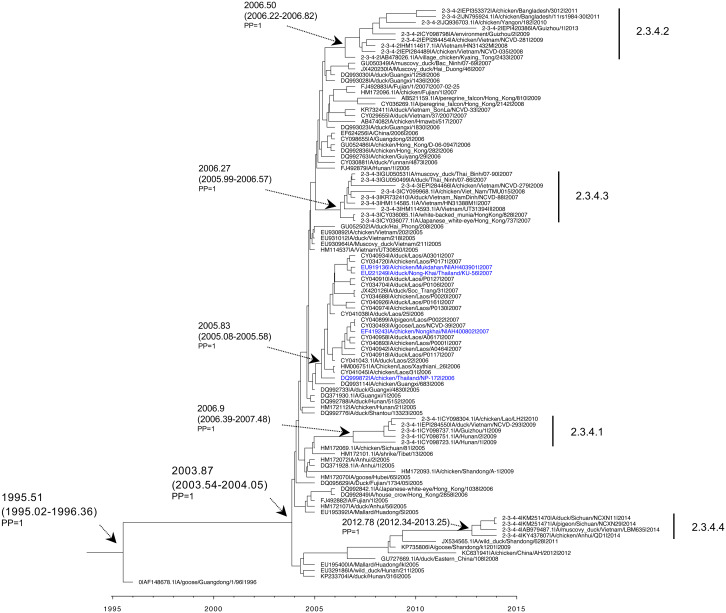
Maximum clade credibility tree based on HA of H5N1 clades 2.3.4, 2.3.4.1, 2.3.4.2, 2.3.4.3, and 2.3.4.4 viruses. Time-scale tree was estimated in BEAST package v1.10.4 with GTR model, strict clock, and exponential growth tree prior. The tMRCA and 95% HPD are indicated with a black arrow, and posterior probability (PP) value is indicated. The name of each taxon is presented in order of GenBank accession number, virus name, and year of collection.

Datasets of HA and NA H5N1 clade 1 and clade 2.3.4 Thailand viruses, H5N1 viruses from neighboring countries, and the Gs/GD/1/96 (H5N1) ancestor strain, were retrieved for Bayesian phylogenetic analysis. An MCC reconstructed tree based on H5N1 HA clade 1 viruses revealed that the rooted tMRCA was 1990.35 (95% HPD: 1987.91–1992.77) with posterior probability (PP) = 1. Subsequently, the H5N1 clade 1 descendant viruses of Gs/GD/1/96 in the neighboring regions such as Hong Kong between 2002 and 2006 had an estimated tMRCA of 1999.07 (95% HPD: 1998.02–2000.18) with PP = 1. The H5N1 clade 1 Thailand viruses clustered together with H5N1 clade 1 viruses from Vietnam, Cambodia, Laos, and Malaysia circulating in 2003–2005. Those viruses formed the monophyletic clade with a PP of 1 by the tMRCA, which was 2000.95 (95% HPD: 2000.41–2001.57). The introduction time of most H5N1 viruses circulating in Thailand from 2003 to 2010 was estimated to be 2001.87 (95% HPD: 2001.34–2002.49). However, the PP support was poor, possibly due to the low number of sampled viruses (Fig. 3). Additionally, the MCC reconstructed tree based on HA revealed that the tMRCA of H5N1 clade 2.3.4 viruses was 2003.87 (95% HPD: 2003.54–2004.05) with PP = 1. The viruses were detected earlier in China and Hong Kong in 2005. The H5N1 clade 2.3.4 Thailand viruses isolated in 2007 were phylogenetically related to viruses from Laos and Vietnam isolated in 2006-2007, showing the monophyletic clade. These viruses shared a tMRCA of 2005.83 (95% HPD: 2005.08–2005.58) with PP = 1 (Fig. 4).

**Figure 5:**
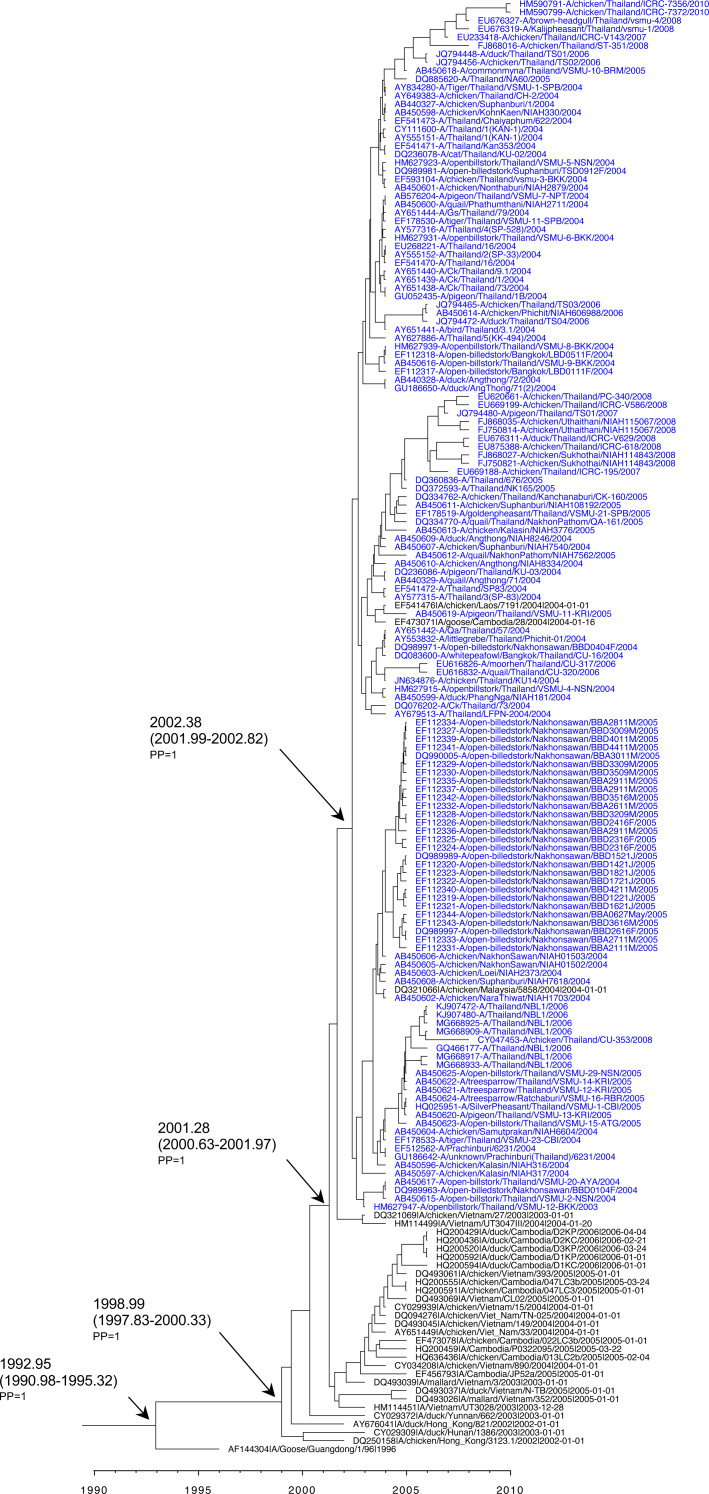
Maximum clade credibility tree based on NA of H5N1 clade 1 viruses. Time-scale tree was estimated in BEAST package v1.10.4 with GTR model, strict clock, and exponential growth tree prior. The tMRCA and 95% HPD are indicated with a black arrow, and posterior probability (PP) value is indicated. The name of each taxon is presented in order of GenBank accession number, virus name, and year of collection.

**Figure 6:**
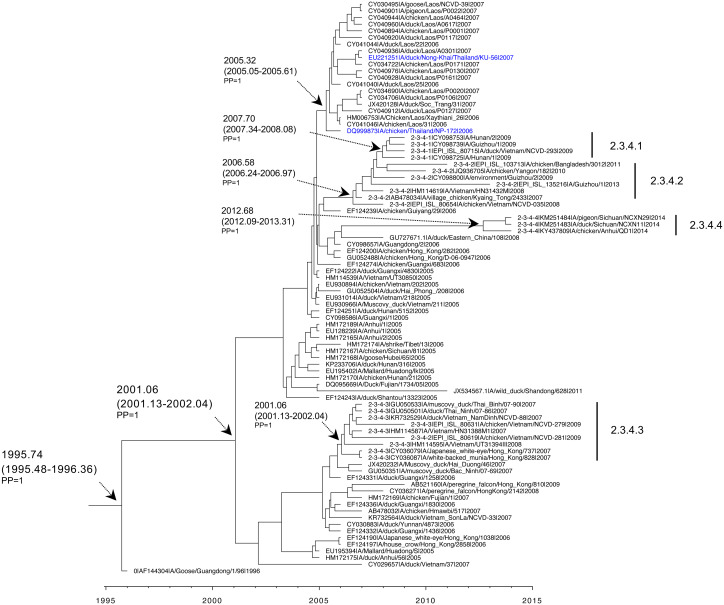
Maximum clade credibility tree based on NA of H5N1 clades 2.3.4, 2.3.4.1, 2.3.4.2, 2.3.4.3 and 2.3.4.4 viruses. Time-scale tree was estimated in BEAST package v1.10.4 with GTR model, strict clock, and exponential growth tree prior. The tMRCA and 95% HPD are indicated with a black arrow, and posterior probability (PP) value is indicated. The name of each taxon is presented in order of GenBank accession number, virus name, and year of collection.

The MCC reconstructed tree based on NA of H5N1 clade 1 viruses revealed that rooted tMRCA was 1992.95 (95% HPD: 1990.98–1995.32) with PP = 1. The NA of Gs/GD/1/96 virus introduced into neighboring regions, (particularly Hunan, Yunnan, and Hong Kong) was around 1998.99 (HPD: 1997.83–2000.33) with PP = 1. H5N1 clade 1 viruses emerged later in Southeast Asia, indicated by the estimated tMRCA of 2001.28 (95% HPD: 2000.63–2001.97). In addition, the earliest of H5N1 Thailand viruses was isolated from openbill stork in 2003 in the central part of Thailand, and then rapidly spread as shown in the short branch. As shown in the phylogenetic tree, the tMRCA of H5N1 NA Thailand viruses corresponding to H5 clade 1 was 2002.38 (95% HPD: 2001.99–2002.82) with PP = 1, together with the related strains from nearby countries including Cambodia, Laos, and Malaysia reported in 2004 (Fig. 5). Moreover, the MCC reconstructed tree based on NA of H5N1 clade 2.3.4 viruses revealed that these viruses emerged in 2001.06 (95% HPD: 2000.13–2001.04). The introduction of H5N1 NA corresponding to H5 clade 2.3.4 Thailand viruses was estimated to be 2005.32 (95% HPD: 2005.05–2005.61) with PP = 1. Thailand viruses formed a cluster together with Laos and Vietnam viruses with a similar collection date from 2006–2007 (Fig. 6).

### ML phylogenetic analysis of HA between H5N1 clade 2.3.4 Thailand viruses and H5Nx clade 2.3.4.4 viruses

**Figure 7:**
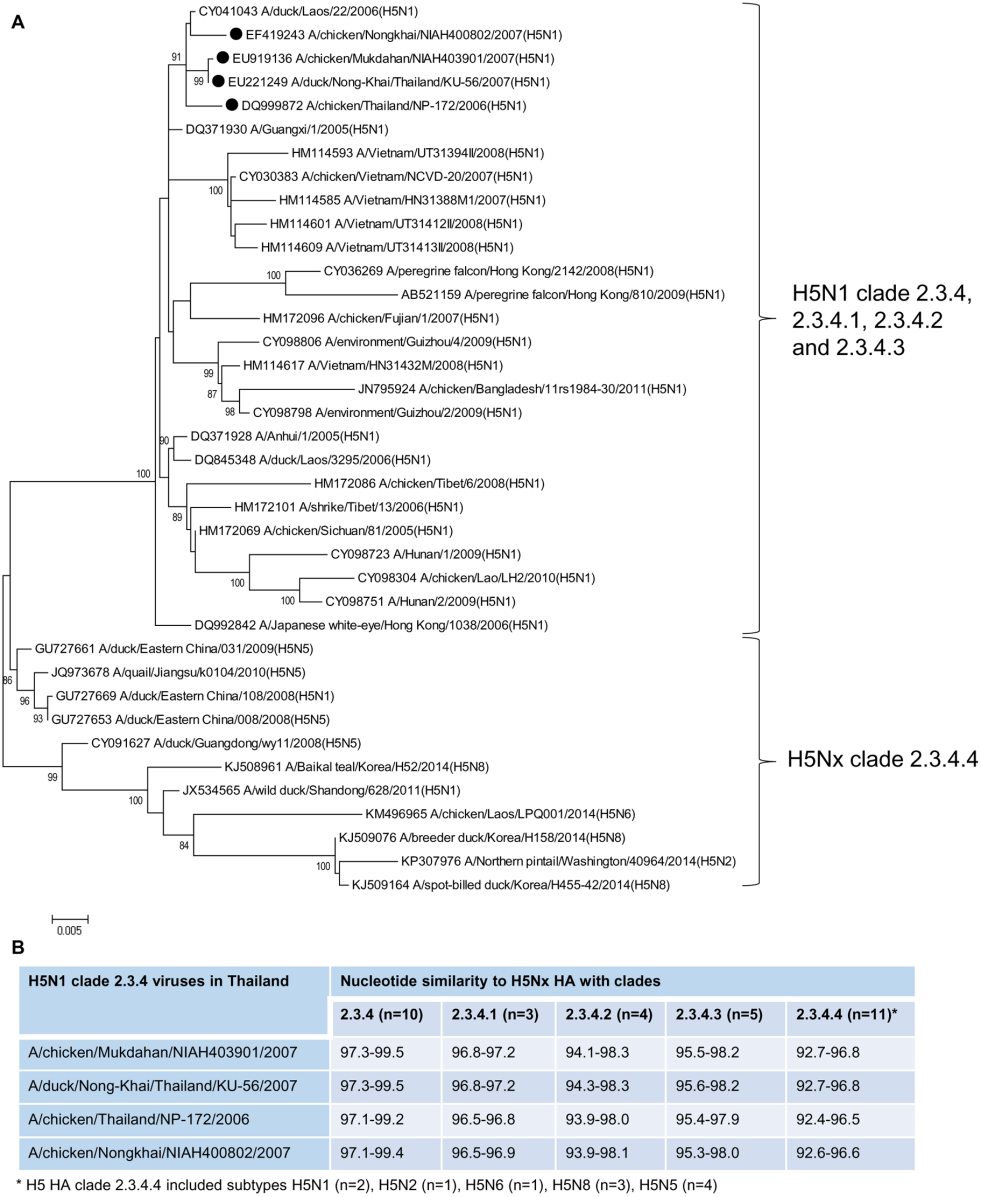
Maximum likelihood phylogenetic analysis (A) and genetic comparison between Thai H5N1 clade 2.3.4 and H5Nx clade 2.3.4.4 (B).

We constructed the ML phylogenetic tree of four H5N1 viruses belonging to clade 2.3.4 (A/chicken/Mukdahan/NIAH403901/2007, A/duck/Nong-Khai/Thailand/KU-56/2007, A/chicken/Thailand/NP-172/2006 and A/chicken/Nongkhai/NIAH400802/2007), and H5Nx clades 2.3.4, 2.3.4.1, 2.3.4.2, 2.3.4.3 and 2.3.4.4 reference strains. The tree topology demonstrated that H5N1 clade 2.3.4 Thailand viruses were closely related to clades 2.3.4, 2.3.4.1, 2.3.4.2, and 2.3.4.3, and clearly separated from the clade 2.3.4.4 with 100% bootstrapping support (Fig. 7A and Fig. S1). They had nucleotide similarities ranging from 92.4–96.8% and 90.1–94.8% to H5Nx clade 2.3.4.4 and clades 2.3.4.4a-h viruses, respectively (Fig. 7B and Table S3).

## Discussion

H5Nx viruses, which have H5 HA in combination with various NA subtypes, caused global poultry disease outbreaks. These viruses, particularly the H5N1 subtype pose a pandemic threat to humans. A H5N1 virus that caused human infections in Hong Kong in 1997 was a reassortant virus that acquired HA from a Gs/GD/1/96 (H5N1)-like virus, NA from a A/teal/Hong Kong/W312/97(H6N1)-like virus, and internal genes from a A/quail/Hong Kong/G1/97(H9N2)-like virus or A/teal/Hong Kong/W312/97 (H6N1)-like virus (Guan et al., 2002; Puthavathana et al., 2005; Gutiérrez et al., 2009). Also, the Gs/GD/1/96 virus was a progenitor which provided *HA* and *NA* genes for the re-emerged H5N1 viruses that caused outbreaks in East and Southeast Asia in 2003 (Gutiérrez et al., 2009), and for descendant HA clade 2.3.4.4 of currently circulating reassortant H5Nx viruses (Smith et al., 2015; Antigua et al., 2019). Thailand first reported H5N1 virus infection in both poultry and humans in January 2004. The first laboratory-confirmed human case occurred in Kanchanaburi province on January 23rd, 2004 (Puthavathana et al., 2005), and the most recent human case was confirmed in September 2006 in Nong Bua Lum Phu province in Northeast Thailand (Sangsiriwut et al., 2021). The causative H5N1 viruses belonged to genotype Z or clade 1 viruses, which subsequently became the predominant viruses circulating in poultry in the central and lower-north region (Uchida et al., 2008; Chaichoune et al., 2009; Amonsin et al., 2010). All human cases in Thailand were infected with the clade 1 virus (Puthavathana et al., 2005; Sangsiriwut et al., 2021). However, genotype V or the clade 2.3.4 virus caused the poultry outbreaks in northeast provinces during 2006 to 2007 (Chutinimitkul et al., 2007).

*HA* and *NA* genes, which encode major viral surface glycoproteins, are hypervariable and continuously evolve (Gutiérrez et al., 2009). The biological properties and immune responses against HA and NA glycoproteins of these H5N1 viruses have been previously characterized (Panaampon et al., 2012; Noisumdaeng et al., 2013; Noisumdaeng et al., 2014; Changsom et al., 2016; Noisumdaeng et al., 2021). This study comprehensively analyzed the genetic evolution of Thai H5N1 HA and NA nucleotide sequences available in the GenBank database from 2003 to 2010. No newer H5N1 data from Thailand were available. ML phylogenetic analysis demonstrated that two clades, the predominant clade 1 and clade 2.3.4, were found in Thailand, corresponding to several previously reports (Uchida et al., 2008; Chaichoune et al., 2009; Amonsin et al., 2010). The replacement of clade 1 with clade 2.3.4 viruses was observed in Southeast Asian countries (Wan et al., 2008; Gutiérrez et al., 2009). Moreover, genetic characterization demonstrated that all H5N1 Thailand viruses were HPAI regarding the presence of multiple basic amino acids (81% of viruses possessed RE RRRKKR↓GLF) at proteolytic cleavage sites on HA. These viruses showed an avian receptor preference by presenting 190E, 225G, 226Q, and 228G (H3 numbering) (Long et al., 2019; Centers for Disease Control and Prevention, 2012). Nevertheless, probable human-to-human transmission was first reported in Thailand (Ungchusak et al., 2005). Additionally, H5N1 NA of all Thailand viruses had a 20-amino acid deletion at the stalk region, which contributed to the high pathogenicity and host adaptation of the virus (Li et al., 2014; Stech et al., 2015). The presence of histidine at amino acid position 275 (N1 numbering) or 274 (N2 numbering) in NA suggested that all viruses were oseltamivir sensitive. Gene-constellation analyzes revealed the intra-H5N1 clade 1 reassortment in poultry (Chaichoune et al., 2009; Amonsin et al., 2010), and one human case (Sangsiriwut et al., 2021).

Our Bayesian phylogenetic analysis of HA revealed that H5 HA viruses emerged approximately in 1990, although the first isolate was reported in 1996 in China (putative ancestor strain Gs/GD/1/96). The viruses circulated among avian host species until 1999 and subsequently re-emerged in 2003. Our analysis postulated that H5 HA clade 1 viruses had an emergence time around 1999, but they were discovered in several cities in China and Hong Kong beginning in 2002. Similarly, the virus strain A/openbilledstork/Thailand/VSMU-12-BKK/2003 (H5N1) (GenBank accession no. HM627945) was the first isolate reported in Thailand, but it may have been introduced into Thailand in 2001. Several reports revealed that the H5 HA clade 1, which predominantly circulated in Cambodia, Thailand, and Vietnam during 2003–2005, was responsible for human infections (Gutiérrez et al., 2009). Likewise, the Bayesian phylogenetic analysis of NA demonstrated that the tMRCA estimation was comparable to the HA phylogenetic tree. The mean rate of nucleotide substitution of *HA* and *NA* genes among clade 1 viruses was 2.46 ×10^−3^ substitution/site/year and 2.47 ×10^−3^ substitution/site/year, respectively (Suwannakarn et al., 2009).

As the result of genetic evolution, the H5 HA clade 2 viruses emerged and continually circulated in several regions in Asia (East, Southeast, and Middle East), Europe and Africa. Subsequently, the clade 2 viruses genetically diverged into distinct subclades and sub-subclades (World Health Organization , 2014; Smith et al., 2015). Thailand reported four isolates of clade 2.3.4 (A/chicken/Mukdahan/NIAH403901/2007, A/duck/Nong-Khai/Thailand/KU-56/2007, A/chicken/Thailand/NP-172/2006, and A/chicken/Nongkhai/NIAH400802/2007), which were isolated to the northeast region of the country. Those viruses were previously reported as genetically related to the Fujian-like virus clade 2.3.4 (Smith et al., 2006; Suwannakarn et al., 2009). Our results revealed that Thailand H5 HA clade 2.3.4 viruses possibly emerged in late 2005. Later, clades 2.3.4.1, 2.3.4.2, 2.3.4.3, and 2.3.4.4, and the proposed update clades 2.3.4.4a-h viruses were identified in several regions in Asia, Middle East, and Europe, but those were not detected in Thailand (World Health Organization , 2014; Smith et al., 2015; World Health Organization , 2021b).

At present, H5Nx clades 2.3.4.4 viruses globally spread and cause outbreaks among wild birds and poultries. H5Nx viruses are likely to become predominant and replace H5N1 viruses in the future. Reassortment has long been known as the major mechanism of viral emergence. HPAI viruses that jump across species to infect humans have emerged through this mechanism. The first emergence of H5N1 viruses occurred in Hong Kong in 1997, followed by the re-emergence of new H5N1 reassortment in 2003, and the emergence of H5N6 and H5N8 subtypes (Li et al., 2017; Antigua et al., 2019; Nuñez & Ross, 2019; Liang et al., 2021; Li, Su & Smith, 2021; Gu et al., 2022). The emergence of these reassortants suggested that the genetic co-evolution of HA and NA through natural genetic reassortment among avian influenza viruses might generate novel pathogenic reassortants (World Health Organization Global Influenza Program Surveillance Network, 2005). Even though the other avian influenza reassortant subtypes (*e.g.*, H7Nx) can cause serious disease in humans, our study was confined solely to the H5 virus subtype. Laboratories worldwide should carry out the monitoring and surveillance of novel avian influenza viruses through genetic analysis for health and safety.

## Conclusion

*HA* and *NA* genes continue to evolve. As such, the reassortant H5Nx viruses generated from reassortment among pools of avian influenza genomic segments presented in avian species. Our results provide information for a better understanding of genetic evolution and molecular epidemiology, as well as support the need for continuous monitoring and active surveillance of H5N1 and H5Nx viruses.

##  Supplemental Information

10.7717/peerj.14419/supp-1 Supplemental Information 1 H5N1 HA dataset analyzed in the study

10.7717/peerj.14419/supp-2 Supplemental Information 2 H5N1 NA dataset analyzed in the study

10.7717/peerj.14419/supp-3 Supplemental Information 3 HA nucleotide similarities between Thai H5N1 clade 2.3.4 and H5Nx clade 2.3.4.4a-h

10.7717/peerj.14419/supp-4 Supplemental Information 4 Maximum likelihood phylogenetic analysis for H5 HA clades 2.3.4, 2.3.4.1, 2.3.4.2, 2.3.4.3, and 2.3.4.4(a-h) The percentage of bootstrapping ( >80) in which the associated taxa clustered together is shown on the nodes. Thai H5N1 and reference viruses are shown in blue and black, respectively. The reference clades are shown with sharp symbol (#) in the front of each tip. The ML tree was rooted to A/goose/Guangdong/1/96 (H5N1).

10.7717/peerj.14419/supp-5 Data S1 Additional raw data of H5N1 and H5Nx viruses analyzed in the study

## Additional Information and Declarations

### Data Availability

The following information was supplied regarding data availability: The raw data are available in the Supplementary Files. The raw data show information of H5N1 and H5Nx viruses analyzed in this study.
