# Supplementary material for: Genetic evolution of hemagglutinin and neuraminidase genes of H5N1 highly pathogenic avian influenza viruses in Thailand
Source: PeerJ. 2022 Nov 30;10:e14419. doi: 10.7717/peerj.14419 (PMC9744161; doi:10.7717/peerj.14419)
Supplement: Supplemental Information 3 [file peerj-10-14419-s003.docx]

**Table S3.** HA nucleotide similarities between Thai H5N1 clade 2.3.4 and H5Nx clade 2.3.4.4a-h

| **H5N1 clade 2.3.4 viruses in Thailand** | **Nucleotide similarity to H5Nx HA with subclade 2.3.4.4** | | | | | | | |
| --- | --- | --- | --- | --- | --- | --- | --- | --- |
|  | **2.3.4.4a (n=3)** | **2.3.4.4b**  **(n=22)** | **2.3.4.4c**  **(n=7)** | **2.3.4.4d**  **(n=3)** | **2.3.4.4e**  **(n=4)** | **2.3.4.4f**  **(n=1)** | **2.3.4.4g**  **(n=4)** | **2.3.4.4h**  **(n=7)** |
| A/chicken/Thailand/NP-172/2006  (GenBank accession no. DQ999872) | 93.1-93.4 | 91.5-94.5 | 92.3-93.0 | 92.4-92.7 | 92.0-93.1 | 93.1 | 90.6-93.3 | 90.1-91.7 |
| A/duck/Nong-Khai/Thailand/KU-56/2007  (GenBank accession no. EU221249) | 93.6-93.8 | 91.7-94.8 | 92.6-93.3 | 92.8-93.1 | 92.3-93.3 | 93.4 | 91.0-93.4 | 90.1-92.0 |
| A/chicken/Mukdahan/NIAH403901/2007  (GenBank accession no. EU919136) | 93.6-93.8 | 91.7-94.8 | 92.6-93.3 | 92.8-93.1 | 92.3-93.4 | 93.4 | 91.0-93.4 | 90.1-92.0 |
| A/chicken/Nongkhai/NIAH400802/2007  (GenBank accession no. EF419243) | 93.1-93.4 | 91.6-94.6 | 92.4-93.0 | 92.4-92.7 | 92.1-93.0 | 93.1 | 90.9-93.2 | 90.1-91.9 |
