# Supplementary figures and images for: Genetic evolution of hemagglutinin and neuraminidase genes of H5N1 highly pathogenic avian influenza viruses in Thailand
Source: PeerJ. 2022 Nov 30;10:e14419. doi: 10.7717/peerj.14419 (PMC9744161; doi:10.7717/peerj.14419)

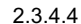

### 2.3.4.2

### 2.3.4.3

#### 2.3.4.1

Supplement: Supplemental Information 4 — The percentage of bootstrapping ( >80) in which the associated taxa clustered together is shown on the nodes. Thai H5N1 and reference viruses are shown in blue and black, respectively. The reference clades are shown with sharp symbol (#) in the front of each tip. The ML tree was rooted to A/goose/Guangdong/1/96 (H5N1). [file peerj-10-14419-s004.pdf]
